# Comparing species richness, functional diversity and functional composition of waterbird communities along environmental gradients in the neotropics

**DOI:** 10.1371/journal.pone.0200959

**Published:** 2018-07-20

**Authors:** Bia de Arruda Almeida, Andy J. Green, Esther Sebastián-González, Luiz dos Anjos

**Affiliations:** 1 Programa de Pós-Graduação e Ecologia de Ambientes Aquáticos Continentais, Universidade Estadual de Maringá, Maringá, Paraná, Brazil; 2 Department of Wetland Ecology, Doñana Biological Station, EBD-CSIC, Sevilla, Spain; 3 Departamento de Biología Aplicada, Universidad Miguel Hernández, Elche, Alicante, Spain; 4 Departamento de Biologia Animal e Vegetal, Centro de Ciências Biológicas, Universidade Estadual de Londrina, Londrina, Paraná, Brazil; Wageningen Universiteit, NETHERLANDS

## Abstract

Waterbirds have a major functional role in wetlands, and understanding how functional traits of waterbirds depend on environmental characteristics can facilitate management of ecosystems and their services. We investigate how the waterbird community in a Neotropical river-floodplain system responds to environmental gradients, identifying how they affect waterbird species richness, functional diversity (measured as functional dispersion) and functional composition (specific functional traits). We sampled 22 lakes in the Upper Paraná floodplain system in southern Brazil, and modelled avian functional diversity and species richness as a function of environmental variables. Then we used a unified RLQ and fourth-corner analysis to evaluate environment-trait relationships. Waterbird species richness and functional diversity varied according to different environmental variables. Lake area and diversity of aquatic vegetation were associated with avian species richness, while relative abundance of grass and emergent macrophytes and mean and variation of depth were related to functional diversity. Furthermore, changes in functional diversity seemed to be mainly driven by presence of species that depend on perches for foraging (e.g. kingfishers, cormorants, and kites), whose presence was mainly associated with deep water and emergent macrophytes. Nevertheless, changes in functional diversity and functional composition did not depend on exactly the same set of environmental variables, suggesting that trait combinations (e.g. below surface feeders who feed on fish), not only specific traits, are important drivers of the variation in functional diversity between lakes. Given the observed differences in responses of species richness and functional diversity, both these diversity metrics should be used as complementary tools in ecosystem management. Furthermore, our results show that functional diversity and composition are partially coupled, suggesting that although functional diversity is influenced by the environmental filtering of particular traits, it also reflects other ecological mechanisms (e.g. competitive interactions among species).

## Introduction

Wetlands provide key ecosystem services such as fishery maintenance, water quality improvement, nutrient fixation, carbon management, and flood prevention [1]. The maintenance of these globally important services depends on physical and chemical processes, but also on biological processes sustained by different groups of organisms within aquatic ecosystems [2]. Waterbirds have a major functional role in aquatic ecosystems, and may have profound effects e.g. through predation [3], herbivory [4], bioturbation [5], guanotrophication [6] and as dispersal vectors [7]. The ways in which waterbirds use resources in wetlands influence their roles in the ecosystem [8]. However, we know little about how the use of resources by the community varies according to particular characteristics of wetlands.

Understanding how waterbird functional traits depend on wetland characteristics is important if we are to maintain functions performed by waterbirds, and consequently ecosystem services. Functional traits are any morphological, physiological or phenological characteristics measurable at the individual level [9], which are linked to ways in which organisms interact with the ecosystem. There are many previous studies of wetland features associated with the habitat use [10] or species richness [11,12] of waterbirds, or with the distribution of particular species [13]. However, taxonomic measures of diversity such as species richness do not account for the diversity of functional traits in the community. For this reason, taxonomic and functional measures of diversity may vary independently, according to different variables. So far, few studies have addressed the effect of environmental variables on the functional traits and functional diversity of waterbird communities [14–16].

There are two different approaches to evaluating community functional structure in an ecosystem. One focuses on the functional diversity of the community, represented by a diversity index that quantifies the distribution of the functional traits of a group of organisms within an ecosystem [17].The other approach focuses on functional composition, and considers individual changes in each of the traits displayed by the species in the community [18]. In this study, we use both approaches to enable a more complete view of how environmental characteristics of floodplain lakes affect functional traits of waterbirds. Measuring functional diversity is essentially quantifying the spread of points (species) in *n*-dimensional trait space [19]. Accounting for functional composition (as determined by particular functional traits) and assessing the relationships between species traits and environmental characteristics allows for a better understanding of which environmental characteristics act as filters. This analysis can indicate which are the habitat characteristics that select particular functional trait values (e.g. higher water depths associated with species that swim underwater).

Both functional diversity and functional composition are important for the maintenance of ecosystem functioning [18,20]. However, most studies focus on only one of these facets of community functional structure, and the relationship between them is rarely explored. Variation in functional diversity may be a product of directional changes in functional composition, through the selection of specific traits by environmental constraints. If this is the case, diversity and composition will respond similarly to environmental variation. Another possibility is that functional diversity and composition are uncoupled, so that different environmental variables determine each aspect of community functional structure [21].

In this study, we investigate how waterbirds in a river-floodplain system in southern Brazil respond to natural environmental gradients related to terrestrial and aquatic vegetation, water transparency, and depth, size and shape of floodplain lakes. We aim to establish how natural environmental variation affects waterbird functional diversity and each functional trait. We use the functional dispersion index (i.e. the dispersion of the traits of the community in the functional space) as a measure of functional diversity, and analyze trait-environment relationships using RLQ and fourth-corner analyses. First, we ask (i) whether functional diversity responds in the same way as species richness to environmental variation, i.e. if changes in both metrics are dependent on the same set of variables. Then, we aim to establish (ii) to what extent changes in functional diversity are a result of the pressure of environmental filters on specific avian traits, by investigating if the same environmental variables affect both functional diversity and composition. Furthermore, if functional diversity and composition are coupled, we aim to clarify (iii) which are the species functional traits that change together with functional diversity for waterbirds in floodplain lakes, and (iv) which environmental variables drive these changes.

## Materials and methods

### Study area

**Fig 1:**
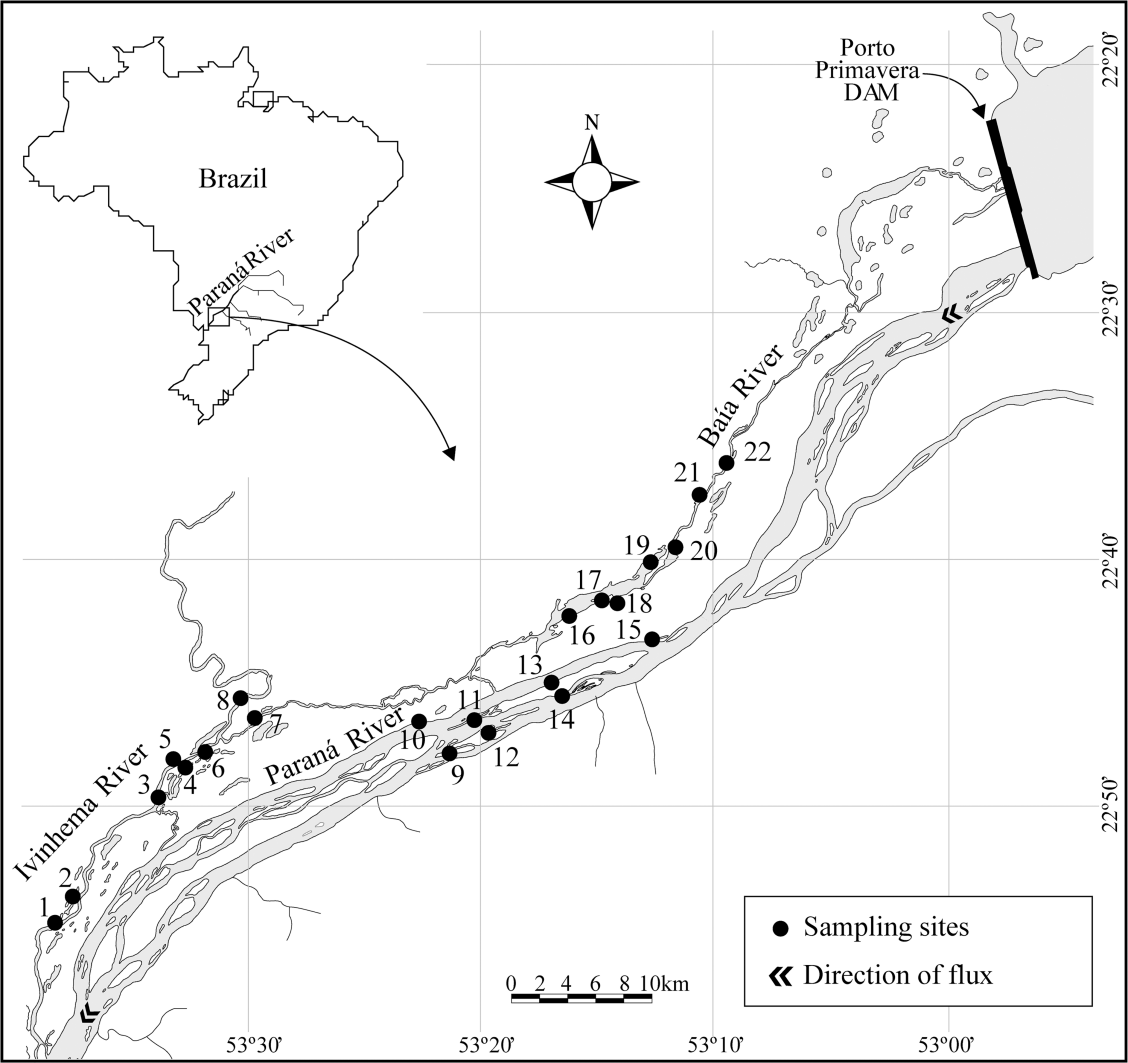
Map of the study area. Numbered points represent the location of the sampled lakes.

The Paraná River is the second longest river in South America. The Upper Paraná River floodplain represents the last remaining undammed stretch of the Paraná River in Brazilian territory. This floodplain stretches for 230 km (22°40’S to 22°52’S and 53°12’W to 53°38’W), between the Porto Primavera and Itaipu reservoirs, the latter being the largest hydroelectric producer in the Americas. The lakes sampled in this study are in the river floodplain between the Paranapanema and Ivinhema tributaries (Fig 1). There is an extensive alluvial plain in the west bank of this section, including the Baía River and the Ivinhema River, and various permanent lakes, which connect to the rivers permanently, or only during floods. The climate is tropical-subtropical, with annual average temperature of 22°C (average temperature of 26°C in summer from December to March and 19°C in winter from June to September). The wet season lasts from October to February, with average rainfall per month exceeding 125 mm, and the dry season from June to September, with an average per month of less than 80 mm. The flooding period on the plain usually occurs from November/December to April/May and is characterized by an average increase in the water level of 2.5 m across the floodplain, reaching 7.5 m in years of extreme flooding events, with little variation in years in which the characteristic flooding period does not occur. The occurrence of two or three pulses per year is common during the floods, whereas smaller pulses (<0.5 m) occur weekly during drought due to the operation of the upstream reservoirs [22]. Lakes sampled in this study were associated with the three main rivers of this section of the floodplain: Paraná, Baía, and Ivinhema. Permission for developing activities in lakes associated to the Ivinhema River was given by the Ivinhema State Park. No permission was required for research activities in lakes associated to the Baía and Paraná Rivers, as these are protected areas with free access. No organism was manipulated during this study. Lakes with both permanent surface connection to the rivers (n = 12), and connected only during flood events (n = 10), were surveyed.

### Bird surveys

Birds were surveyed in 22 lakes (mean distance between lakes 21.4 km ± 13.9, range: 0.6–58.7 km) three times: from March 18^th^ to April 5 ^th^, from September 22 ^th^ to October 10 ^th^ in 2014, and from 7 ^th^ to 20 ^th^ April in 2015 (Fig 1). Campaign dates were set in order to avoid flood or extreme drought periods in the floodplain, so as to represent the conditions that occur during most of the year. Moreover, during periods in which there is no flood, environmental heterogeneity is maximized, exposing environmental gradients and allowing the test of related hypotheses [23]. We established two sampling periods, one in the morning lasting four hours and beginning one hour after sunrise, and one in the afternoon lasting three hours and ending one hour before sunset. In each campaign, each lake was surveyed three times by the same observer, during different sampling periods on the same day or on different days (i.e. each lake was surveyed nine times in total). We made several surveys each day, always resampled the same lake at different times of the day, and never resampled the same lake twice in the same day *and* period.

We surveyed lakes using a transect-sampling method along their margins, in which each individual sighting was recorded [24]. Surveys were conducted by boat in all lakes, except for four of them, which were sampled on foot. The duration of the survey depended on the time required to go through the entire transect, identify and count all the waterbirds on the lake, and ranged from 20 to 50 minutes. All lakes were sampled along the entire margin. Individual sightings of birds up to 5 m away from the water edge were included, including birds perched in trees or other vegetation. Birds in flight were only counted if they were observed leaving the lake or landing in it. All surveys were made by the same observer with 10 x 50 binoculars, and particular care was taken to ensure that individuals were only counted once. The abundance of each species per survey was the number of individuals recorded. For each species, the survey with the highest number of individuals recorded was considered to represent the total abundance for a given lake (see [24]). The total number of species found in at least one survey was used as total species richness for a given lake. Birds included in this study were all of those that feed in or on water, including those families defined as waterbirds by Wetlands International [25], plus three members of the Accipitridae family (S1 Table). We used the American Ornithologists’ Union nomenclature for species [26].

### Environmental variables

We recorded 16 environmental variables for each lake (Table 1). These variables have been shown in previous studies to be important in determining the use of wetlands by waterbirds [10]. First, we classified terrestrial vegetation into three types (tree, bush, and grass) and aquatic vegetation into four types (floating, emergent, a mixture of floating and emergent, and absence of aquatic vegetation). The tree and bush vegetation types were separated by height, with trees having height > 2 m. Then, we estimated the proportion of the lake margin covered by each type of terrestrial and aquatic vegetation. Predominant marginal vegetation types (both terrestrial and aquatic) were determined by the observer every 10 seconds during the transect around the lake. The proportion of the lake margin covered by each type of vegetation (*P*) was then calculated according to the number of observations classified as a vegetation type (*x*) in the total number of observations (*n*) as in: P=xn. This procedure was repeated three times in each campaign for each lake, totaling nine samplings per lake. We used the mean of the proportion calculated for the nine samplings as our measure of margin occupation by each vegetation type in each lake. Finally, we calculated an index of diversity of vegetation types in each case (separately for terrestrial and aquatic vegetation types) by combining the proportions found for all types of vegetation, according to the equation in Table 1. Vegetation variables were quantified during bird surveys.

**Table 1:** Environmental variables recorded for sampled lakes and their definition.

Environmental variable code	Environmental variable definition
Tree	Proportion of tree type of terrestrial vegetation occupying the lake margin
Bush	Proportion of bush type of terrestrial vegetation occupying the lake margin
Grass	Proportion of grass type of terrestrial vegetation occupying the lake margin
TVdiv	Diversity index for the three terrestrial vegetation types as in $D=1/\sum_{i-1}^{n}p_{i}^{2}$
Floating	Proportion of floating type of aquatic vegetation occupying the lake margin
Emergent	Proportion of emergent type of aquatic vegetation occupying the lake margin
Both	Proportion of both floating and emergent types occurring together in lake´s margin
NoVeg	Proportion of lake’s margin without aquatic vegetation
AVdiv	Diversity index for the four considered aquatic vegetation types as in $D=1/\sum_{i-1}^{n}p_{i}^{2}$
Transparency	Water transparency measured in meters with a Secchi disk
Mdepth.center	Mean of three depth measurements in the center of the lake
VCdepth.center	Variance coefficient of three depth measurements in the center of the lake
Mdepth.margin	Mean of 10 depth measurements in the margin of the lake
VCdepth.margin	Variance coefficient of 10 depth measurements in the margin of the lake
Area	Lake area in hectares, measured from Google Earth images
Prop.per.area	Ratio between lake’s perimeter and area

*p_i_* = margin proportion occupied by *i*-th vegetation type. n = number of vegetation types

We measured water transparency and depth at the lakes once in each campaign. Transparency was measured with a Secchi disk in three points distributed along a straight transect in the central area of the lake. A mean of the total of nine measurements (three points in each campaign) was used as the final Transparency value. Depth was measured separately once in each campaign for lake margins and the central area. For the margins, depth was measured at ten points right at the edge of macrophyte beds. For the center of the lake, it was measured in the same three points where transparency was measured. From these depth measurements we calculated the mean and variance coefficient separately for the margin and center of the lakes. The area and perimeter of each lake were measured from Google Earth images. See Table 1 for environmental variable codes and definitions.

### Functional traits

We measured functional traits of waterbirds related to resource use, as birds perform most of their ecological roles via resource acquisition [27,8]. Thus, we used 22 functional traits related to variation in the birds’ ability to exploit resources (See Table 2 for trait codes and definitions and S2 Table for traits of each species used for analyses). Binomial traits were sourced from Del Hoyo et al. [28], and other traits from Wilman et al. [29].

**Table 2:** Waterbird functional traits used in this study.

Functional trait code	Functional trait definition
Body mass	Body mass in grams
Invertebrates	Percentage of diet composed of invertebrates
Endotherms	Percentage of diet composed of endotherm vertebrates
Ectotherms	Percentage of diet composed of reptiles and amphibians
Fish	Percentage of diet composed of fish
Vertebrates	Percentage of diet composed of vertebrates of unknown group
Scavenge	Percentage of diet composed of carrion
Fruits	Percentage of diet composed of fruits
Seeds	Percentage of diet composed of seeds
Plant material	Percentage of diet composed of other plant material
Diet plasticity	Number of items present in diet
Below surface	Percentage of use of water below surface feeding stratum
Around surface	Percentage of use of water around surface feeding stratum
Ground	Percentage of use of ground feeding stratum
Understory	Percentage of use of understory feeding stratum
Mid-high	Percentage of use of mid-high feeding stratum
Strata plasticity	Number of strata used in food acquisition
Long legged	Binomial, for presence of legs longer than body
Hooked bill	Binomial, for presence of hooked bill
Long bill	Binomial, for presence of bill longer than head
Swim	Binomial, for ability to swim
Perch	Binomial, for use of perch for foraging

Traits were sourced from Del Hoyo et al. [28] and Wilman et al. [29].

Body mass strongly relates to metabolic rate, indicating the amount and size of food required for a given individual [30]. Percentages of diet composition and stratum use indicate the main items consumed by species, and where they are acquired, and thus are related to avian functions such as population control, propagule dispersal, scavenging, nutrient cycling, and ecosystem engineering [8]. Plasticity of diet and strata are continuous measures that represent the level of specialization associated with a given species. Higher values for both plasticities indicate less dependence on a particular food item or stratum [31]. The binomial traits used in this study indicate morphological and behavioral characteristics associated with how bird species procure food. Five such traits were used, three indicating morphological adaptations (long legged, hooked bill, and long bill) and two behavioral adaptations (swim and perch). Long legged birds acquire resources by walking or wading, and thus need substrate for standing while foraging. Bill shape is a morphological adaptation related to the way in which food is accessed. Finally, perch and swim are behavioral traits that indicate the need that a species has for a substrate to perch on, and its ability to swim, and thus not being completely dependent on shallow water or a substrate near the water to be able to forage.

### Functional diversity

We calculated the index functional dispersion (FDis; [32]) as a representative of functional diversity for each sampled lake. FDis is a multivariate measure of the dispersion of species in the trait space and represents the mean distance of species to the centroid of the community, weighted by their abundances. Functional dispersion, as functional richness, is a measure of the dispersion of species in the functional space. However, it is independent of species richness by construction, so that its use ensures that the number of species does not influence the response of functional diversity to environmental gradients. Thus, we use FDis because it is an intuitive measure of functional diversity that accounts for both the volume of occupied functional space and the distribution of species within this space. In order to avoid an over-representation of the variables related to diet and feeding strata in the calculation of functional diversity, we reduced their weight so that the weight of all the ‘diet items’ columns and all the ‘feeding strata’ columns respectively was equivalent to one of the columns for the other variables. We computed the functional distances between pairs of species using the Gower distance [33]. The Gower distance is deemed appropriate as it can deal with both categorical and continuous traits, missing trait values, and weighting of traits [34,35]. Then, we performed a PCoA (Principal Coordinates Analysis) [33] on the functional distance matrix and used the PCoA axes to represent new trait values. Finally, we calculated FDis from these new trait values and from the abundance data for each species [32]. We calculated FDis with the function dbFD from the FD package [32] in R [36,37]. We also calculated Functional Richness, the volume occupied by the community in the functional space (FRic; [38]), and related it to environmental variables, but the results were very similar to those found for Species Richness (SR), and are not presented.

### Statistical analyses

To examine the relation between environment and functional dispersion, we constructed beta regression models using our environmental variables as explanatory variables and FDis as a response variable. Beta regression is appropriate for dealing with continuous response variables that are restricted between zero and one, as is the case of FDis [39]. Before model construction, we evaluated pairwise correlations among explanatory variables using Spearman’s rank correlation coefficients (r) to avoid multi-collinearity. Tree proportion was then removed from models, since it had a high correlation with bush and grass proportions (|r| >0.7). We also evaluated if there was a spatial autocorrelation in our data, but Moran’s I tests indicated a lack of it for SR (Moran´s I: 0.019, p-value: 0.45) or FDis (Moran’s I: 0.115, p-value: 0.07). We used log_10_ transformation for continuous variables and arcsine√(x) for variables expressed in proportion (vegetation proportions) to reduce data dispersion and improve linearity. We applied a model-averaging approach that accounts for model uncertainty, increases the robustness of the parameter estimates, and assesses the relative importance or each of the predictor variables [40,41]. Model-averaging started with a global model with all the environmental variables previously described, except for tree proportion, fitted using the lme4 package in R [42]. Then, we used the dredge function of the MuMIn package [43] to create a set of models with all combinations of variables, and from these we identified our best models based on comparisons of Akaike’s Information Criterion (AIC; [44]) with correction for small sample size (AICc; [45]). Then, we selected the models with ΔAICc ≤ 2, which were considered equally plausible. We then produced averaged parameter estimates from this set of selected models and we calculated the relative importance of each variable using the model.avg function. The relative importance was calculated through the sum of Akaike weights across all the selected models, with a weight of zero for models where a given parameter was absent [40]. In addition, we calculated the pseudo r^2^ of the best-selected model (i.e. model with the lowest AICc) as a measure of model fit.

In order to evaluate the response of species richness to the natural environmental gradients in the lakes, and investigate differences between the drivers of richness and functional dispersion, we constructed Generalized Linear Models with a negative binomial error distribution with the same environmental variables, using Species Richness as response variable. We followed the above procedures for environmental variable transformation and model averaging. We calculated the proportion of explained variance of the best-selected model as a measure of model fit.

We also tested the relation between environmental variables and the functional composition of the community using RLQ [46] and fourth-corner analyses [47]. RLQ analysis is useful for exploring the link between multiple environmental variables and multiple species traits, as it allows the exploration of the joint structure of three tables: sites x environmental variables (table R), species x functional traits (table Q), and species x sites (table L). Here, the covariance between tables R and Q is constrained by the abundance of species, present in table L. RLQ analysis selects the axes that maximize the covariance between linear combinations of the columns of tables R and Q [46]. Table R included all environmental variables recorded in our 22 sampled lakes, including the variable Tree, which were transformed as for model constructions. Table Q included all functional traits compiled for the bird species present, i.e. the same traits used to compute FDis. Table L included abundances of species present in our 22 lakes, and underwent a square root transformation before further analysis. According to RLQ protocol, we first analyzed the three tables separately, with different ordination methods. The L table was analyzed with a Correspondence Analysis (CA). The R table was analyzed with a Principal Component Analysis (PCA) using the CA site scores as row weights to couple R and L. The Q table was analyzed with a Hill Smith PCA [48], which combines quantitative and qualitative variables, using the CA species scores as column weights to couple Q and L. The RLQ analysis was then performed to combine the three independent analyses in a single ordination, using the function rlq from the ade4 package [49] in R. The RLQ generated scores were compared to those from separate ordinations to assess how much of their variability was taken into account by the RLQ, and to evaluate the strength of the relationship between traits and environmental variables. Fourth-corner tests were applied to test the significance of correlations between traits, environmental variables, and the first two RLQ axes, as proposed by Dray et al. [47]. Significance was tested using a permutation procedure with the model 6 of the fourthcorner.rlq function, which is a combination of models 2 (permutation of sites) and 4 (permutation of species). We used 9,999 permutations and the false discovery rate method (FDR) to adjust *P* values for multiple testing.

## Results

### Drivers of species richness and functional diversity

A total of 39 waterbird species were recorded in the floodplain lakes (S1 Table). Species richness (SR) per lake ranged from seven to 26 (mean = 13.22, SD = 4.48) and functional dispersion (FDis) ranged from 0.09 to 0.23 (mean = 0.16, SD = 0.04). Correlation between SR and FDis for the waterbird communities was very low (Pearson’s r = 0.04). For SR, model averaging indicated that area and aquatic vegetation diversity were the most important predictors (proportion of explained deviance of the best model = 0.48, Table 3 and S3 Table). Increases in both area and aquatic vegetation diversity led to an increase in number of species per lake (Table 3). Also, while not showing significant confidence intervals, floating and emergent macrophytes had moderate positive and negative effects on SR, respectively. On the other hand, FDis was positively affected by the proportion of the lake margin covered by emergent macrophytes as well as the mean and variation of water depth, but negatively affected by the proportion of margin covered by grass (pseudo r^2^ of the best model = 0.59, Table 4 and S4 Table). Water transparency and absence of vegetation showed moderate negative effects on FDis. Thus, the environmental predictors explaining species richness and functional dispersion were different.

**Table 3:** Model-averaged standardized coefficients (based on models summarized in S3 Table), unconditional standard errors, 95% confidence intervals, and relative importance of environmental predictors of waterbird species richness.

	Standardized coefficient	Unconditional SE	95% CI 2.5% 97.5%		Relative importance of overall predictor
Intercept	1.432	0.585	0.236	2.628	
Area (ha)	0.193	0.070	0.046	0.339	1.00
AVdiv	0.718	0.521	0.030	1.775	0.80
Floating	0.212	0.269	-0.010	0.905	0.47
Emergent	-0.148	0.253	-0.954	0.021	0.32

AVdiv is aquatic vegetation diversity index.

**Table 4:** Model-averaged standardized coefficients (based on models summarized in S4 Table), unconditional standard errors, 95% confidence intervals, and relative importance of environmental predictors of waterbird FDis.

Predictor	Standardized coefficient	Unconditional SE	95% CI 2.5% 97.5%		Relative importance of overall predictor
Intercept	-2.079	0.258	-2.584	-1.574	
Grass	-0.567	0.185	-0.930	-0.204	1.00
Mdepth.margin	0.233	0.062	0.112	0.355	1.00
VCdepth.margin	0.013	0.004	0.004	0.021	1.00
Emergent	0.255	0.223	0.119	0.679	0.64
Transparency	-0.104	0.232	-0.992	0.043	0.22
NoVeg	-0.090	0.201	-0.846	-0.052	0.20

Mdepth.margin = mean margin depth; VCdepth.margin = variation coefficient of margin depth.

### Drivers of functional composition

The first two axes of the RLQ multivariate analysis explained 80.67% of the total inertia of the three tables (Table 5). These axes accounted for most of the variability explained by the first two axes of the separate analyses of environmental variables (R-table) and species functional traits (Q-table), although variability was better explained for environment than traits. Our results indicated a stronger relation between environmental variables and traits on the first RLQ axis. Nevertheless, the correlation between the sets of sites and species scores in RLQ was low for both axes (Table 5).

**Table 5:** Summary of the RLQ analysis.

Total inertia: 0.979			
Projected inertia (%):			
	Ax1	Ax2	
	54.235	26.436	
Eigenvalues decomposition:			
	eig	covariance	correlation
eig1	0.531	0.729	0.230
eig2	0.259	0.509	0.174
Inertia & coinertia R:			
	inertia	max	ratio
eig1	2.927	5.612	0.522
eig1 + 2	8.063	8.646	0.933
Inertia & coinertia Q:			
	inertia	max	ratio
eig1	3.436	4.275	0.804
eig1 + 2	5.094	7.773	0.655
Correlation L:			
	correlation	max	ratio
eig1	0.230	0.515	0.446
eig2	0.174	0.460	0.379

**Fig 2:**
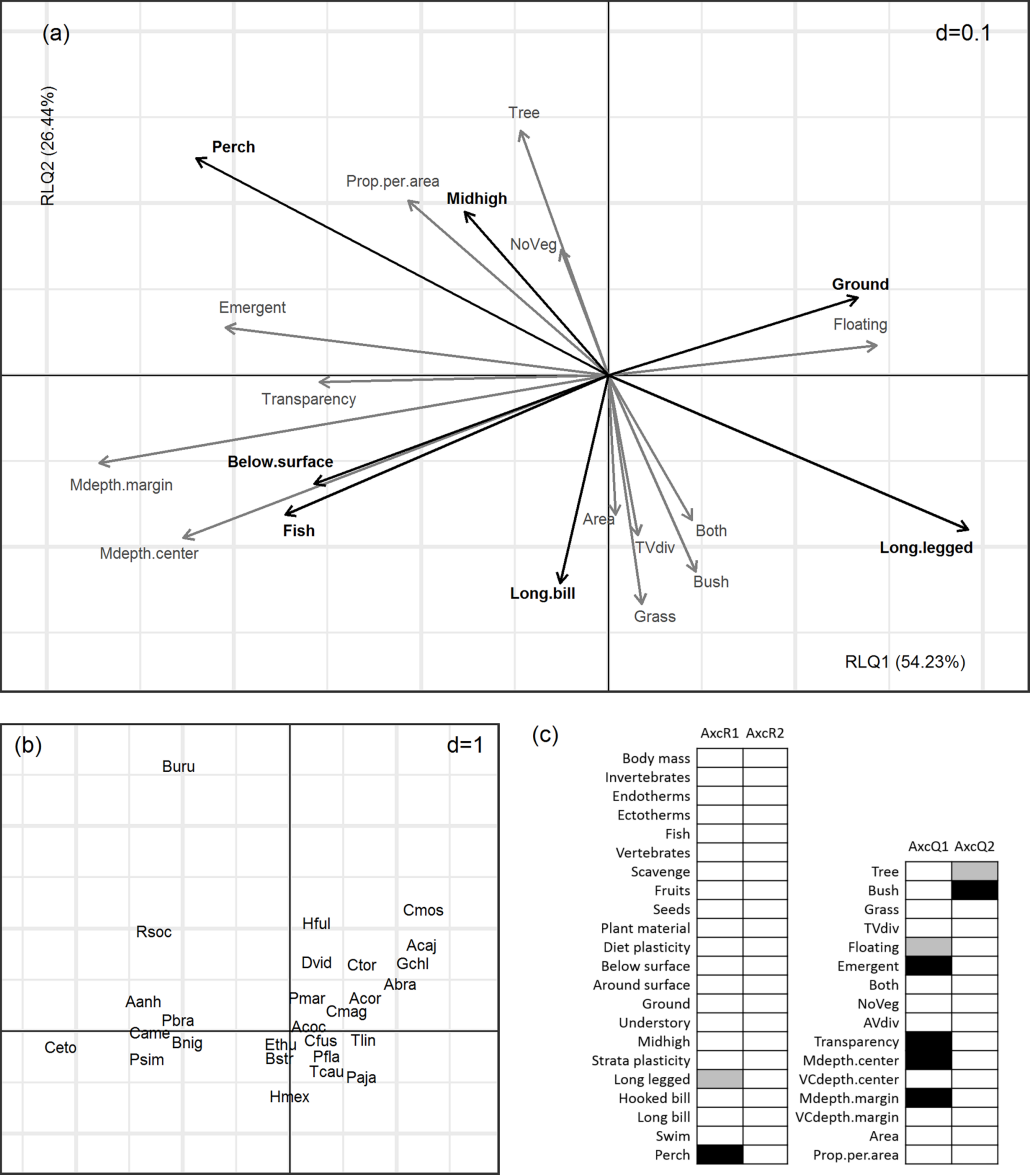
RLQ multivariate analysis. Axes and scale are the same in figures (a) and (b), which represent projections in the plane of the first two main components of: (a) environmental variables and species traits; and (b) waterbird species. Only environmental variables and traits with correlations above 0.6 with at least one of the RLQ axes are represented. Environmental variables are represented in grey and traits in black. See Tables 1 and 2 and S1 Table for environmental variables, functional traits and species that correspond to abbreviations. Species names are centered on the corresponding points, and overlapped species have been removed to ease visualization. Values of *d* give the grid size. Results of the fourth-corner analysis are presented in (c), where grey and black filled squares represent negative and positive correlations respectively.

The joint approach of RLQ and fourth-corner analyses allowed the investigation of the significance of the relations between axes and environmental variables and traits. We found significant negative correlations between the first RLQ axis and the proportion of the margin covered by emergent macrophytes, water transparency, mean margin depth, and mean center depth, and a significant positive correlation between this axis and the proportion of the margin covered by floating macrophytes (Fig 2A and 2C). This axis was significantly correlated to two functional traits, showing a positive association with long legged birds and a negative association with birds that forage from perches (Fig 2A and 2C). Consequently, species that forage from a perch (e.g. kingfishers, cormorants, and kites) were present in lakes with greater depths, higher transparency, and higher cover of emergent macrophytes. On the other hand, species that have longer legs in relation to body size (e.g. jacanas, herons, and storks) were present in lakes with higher cover of floating macrophytes (Fig 2A and 2B). The second axis of the RLQ had a significant positive association with the proportion of trees occupying lake margins, and a significant negative association with the proportion of bushes occupying margins (Fig 2A and 2C). The second RLQ axis showed no significant correlations with traits. A combination of the lower explained variability for traits and the weaker covariance between R and Q tables in the second axis may explain the lack of significance of the associations between traits and the second axis of the RLQ.

## Discussion

We found that waterbird species richness and functional dispersion vary according to different environmental variables in the floodplain, indicating that trait dispersion in the functional space and the number of species are uncoupled in these waterbird communities. To our knowledge, ours is the first study to compare the relationship between environmental variation and species richness and functional diversity for tropical waterbirds. Previous work on terrestrial birds from a tropical arid area showed that species richness and functional differentiation among species showed opposite responses to rainfall and vegetation structure [50]. Furthermore, bird communities from the Amazon forest showed decreased species richness in response to fire events without reduction in functional diversity [51], and bird species richness and functional diversity showed different responses to fragmentation [52]. Overall, multi-trait functional diversity measures can predict functions provided by animal groups better than indices based only on numbers and abundances of species [53]. This indicates that focusing only on species richness is insufficient if we aim to preserve the ecological functions of birds, as there may be a decrease in the diversity of functional traits without a reduction in richness. Bird monitoring and management therefore should include data on both the taxonomic and functional diversity of communities, because they represent complementary information.

### Drivers of species richness

Lake area and diversity of aquatic vegetation were the most important variables explaining species richness. The positive relationship found between lake area and richness is expected according to the species-area relationship [54] and results from previous waterbird studies (e.g. [11,12]). Larger lakes may have higher values of species richness as a product of a sampling effect (i.e. larger lakes can hold more individuals and thus have a higher chance of holding individuals from different species) and/or of higher habitat heterogeneity. One of the many aspects of habitat heterogeneity is the diversity of aquatic vegetation. The relationship found between species richness and the diversity of aquatic vegetation indicates that habitat heterogeneity is indeed important for waterbird SR in this system. This has also been shown by Lorenzon et al. [55] for wetlands associated with this same river. Diverse habitats provide the resources required by a larger number of different species [56]. Thus, our results show that lake area and aquatic vegetation diversity are both important drivers of waterbird species richness.

### Drivers of functional diversity

Mean and variation of depth at the margin, and proportion of the margin covered by grass and emergent macrophytes were the most important variables explaining functional dispersion. The dispersion of traits increased with increasing mean and variation of depth, indicating that lakes with deeper and more variable margins allow a broader distribution of traits in the community. The effect of water depth on the use of wetlands by waterbirds is well known, as it determines the accessibility of foraging habitats according to bird morphology [57–59]. Furthermore, higher variation in margin depth allows different traits to co-exist in the lake, as depth is a determinant of prey accessibility. The consequences of depth variation for functional diversity were also shown by Almeida et al. [15] for wading birds in an earlier dataset from this same floodplain. Moreover, more emergent macrophytes allow a more dispersed distribution of species in the trait space, probably due to the addition of some species that are very dependent on this type of vegetation for foraging and protection [60]. On the other hand, increasing presence of grasses was associated with a decrease in functional dispersion. Grasses are a poor substrate for perching, and are also less valuable as shelter than trees and bushes. Lack of perching sites may limit the access for species possessing certain traits in the waterbird community, reducing trait dispersion. This finding has important implications for the conservation of neotropical waterbirds, as natural vegetation surrounding wetlands in Brazil is progressively being changed into grasslands for cattle grazing [61].

Alterations in functional dispersion are actually changes in the position that individuals have in the space built from functional traits [32]. A higher FDis is a wider distribution of species in the functional space, meaning that more individuals occupy the margins of the functional space in relation to its more central part [21,32]. This occurs because there is a higher number of extreme trait values in the community, which can happen due to increases in abundance or appearance of species with more extreme trait values, or decreases in abundance or disappearance of species with more central trait values. Communities with higher FDis sustain a wider range of ecological traits, suggesting also a wider range of waterbird-mediated ecological functions. Thus, information of the FDis of the community can be highly valuable to understand the ability of the communities to maintain ecological functions under scenarios of species loss.

### Functional composition versus functional diversity

Ours is the first study to analyze in detail the relationship between functional composition and diversity along environmental gradients for avian communities. We found that functional composition and dispersion varied according to different environmental characteristics. Thus, changes in functional dispersion do not depend completely on the pressure of environmental filters on specific traits. This suggests that shifts in composition and dispersion of traits capture signals from different ecological mechanisms, as previously suggested for plants in coastal dunes [62]. Such differences between responses of functional dispersion and composition have also been attributed to trait covariance [63,64]. According to this idea, traits are simultaneously coordinated by environmental variation and, at the same time, respond to this variation through natural selection. This causes multiple potential responses between trait composition and diversity. Functional composition and diversity are both important in different ways for multiple ecosystem processes [65,20]. Our data suggest that these aspects of communities are uncoupled. Therefore, both of them should be taken into account in a common framework when evaluating changes in community functional structure [65].

Only two of the environmental variables affecting functional dispersion and functional composition were shared (mean margin depth and emergent macrophytes), indicating that these two metrics are partially coupled. A decrease in multivariate dispersion in trait space indicates that community composition has shifted towards species that are more similar to each other [66]. If dispersion and composition respond to the same set of variables, a decrease in functional dispersion would mean that the traits driven by this set of variables are being lost, and that this loss causes a decrease in community trait dispersion. This may be the case for decreases in functional dispersion associated with lower lake depths and lower proportion of emergent macrophytes, because both variables negatively affected perching birds. However, for changes in functional dispersion associated with other environmental variables, it seems that the loss of more dissimilar species is not due to loss of a particular trait, as our results show no other trait-environment relationships. Instead, such changes may be caused by the loss of specific trait combinations. Thus, our results suggest that changes in functional dispersion are only partly a result of the pressure of environmental filters on specific traits.

Moreover, only two of the functional traits studied were filtered by our environmental variables: leg length and perching behavior. It seems that changes in functional dispersion are related to the presence of waterbird species that depend on perches as a foraging strategy, and whose presence is mainly associated with higher water depth and the presence of emergent macrophytes. Although emergent macrophytes do not usually provide perches for perching birds, they may influence the accessibility and distribution of prey in the water for these species [67]. Besides, trait combinations, not only specific traits, are important for the variation in functional dispersion between lakes (e.g. below surface feeders which feed on fish), as the location of each species in the functional space depends on values for the entire set of traits. Our data suggest that the species foraging in shallow habitats (mainly long-legged species such as wading birds) are found in most of the lakes. Variation in functional dispersion between lakes then seems to be related to the presence of a minority of species of the regional pool, which depend on active capture of prey in deeper waters.

Variation in the environmental variables of the lakes was low and these variables were linearly related to the functional traits. However, some of these relationships can turn unimodal in areas with a larger variability in the environmental characteristics. Much higher levels of vegetation, for example, could affect negatively the presence of many species of waterbirds, such as wading birds, through foraging difficulties [10]. Also, the type of habitat (e.g. lakes in a floodplain) may determine the dominant traits in the community. Using data from different types of habitat may alter trait response to environmental variation by increasing the ranges of environmental gradients. Another factor influencing our results is the type of environmental variables used. For example, our results could change if we were to investigate environmental variables related to the availability of prey, instead of the accessibility to prey. The presence of bird species with certain traits in the lakes is probably a product of both the availability of prey and of the environmental characteristics that make them accessible for birds [59]. Quantifying both types of variables may therefore have revealed other important relations between traits and environmental gradients. Furthermore, considering distinct facets of functional diversity, not only functional dispersion, could have brought more information on the response of waterbirds to environmental variation [32]. Other indexes of functional diversity add information related to other aspects of functional trait distribution on the functional space.

### Conclusion

In conclusion, our results suggest that different environmental characteristics are important for different aspects of waterbird diversity. Thus, we encourage using distinct ways of measuring diversity (e.g. taxonomic and functional diversity) in ecosystem management. Furthermore, functional diversity and composition are partially coupled, being influenced both by shared and unshared variables. This suggests that functional diversity is indeed influenced by the environmental filtering of particular traits, but that it also reflects other ecological mechanisms, such as competition, which would drive trait divergence. Further investigation of the mechanisms driving the distribution of species traits in other waterbird communities in different biogeographical regions is recommended to test ecological hypotheses of community assembly.

## Supporting information

S1 Table Species found in the sampled area and lakes where they were recorded. (DOCX)

S2 Table Functional trait values for each species used in this study. (DOCX)

S3 Table Top-ranked candidate models explaining variation in waterbird species richness in the floodplain lakes. AICc = Akaike Information Criteria corrected for small sample sizes. Model terms are coded as: 1, Area (ha); 2, AVdiv = aquatic vegetation diversity index; 3, Floating; 4, Emergent. df: Degrees of freedom.
(DOCX)

S4 Table Top-ranked candidate models explaining variation in waterbird FDis in the floodplain lakes. AICc = Akaike Information Criteria corrected for small sample sizes. Model terms are coded as: 1, Emergent; 2, Grass; 3, Mdepth.margin; 4, NoVeg; 5, Transparency; 6, VCdepth.margin (variation coefficient of margin depth). df: Degrees of freedom.
(DOCX)
