# Supplementary material for: Comparing species richness, functional diversity and functional composition of waterbird communities along environmental gradients in the neotropics
Source: PLoS One. 2018 Jul 20;13(7):e0200959. doi: 10.1371/journal.pone.0200959 (PMC6054399; doi:10.1371/journal.pone.0200959)
Supplement: S1 Table — (DOCX) [file pone.0200959.s001.docx]

S1 Table. Species found in the sampled area and lakes where they were registered.

| Taxa | Abbreviation | Common names | | Lakes |
| --- | --- | --- | --- | --- |
| **ANSERIFORMES** |  | |  |  |
| **Anhimidae** |  |  | |  |
| *Anhima cornuta* | Acor | Horned Screamer | | 1, 2, 9, 12, 14 |
| *Chauna torquata* | Ctor | Southern Screamer | | 16, 20, 21 |
| **Anatidae** |  |  | |  |
| *Dendrocygna viduata* | Dvid | White-faced Whistling-Duck | | 17, 20, 21 |
| *Dendrocygna autumnalis* | Daut | Black-bellied Whistling-Duck | | 13, 21 |
| *Cairina moschata* | Cmos | Muscovy Duck | | 2, 5, 8, 9, 11, 12, 13, 14, 19, 22 |
| *Amazonetta brasiliensis* | Abra | Brazilian Teal | | 18, 19, 20 |
| **GRUIFORMES** |  |  | |  |
| **Aramidae** |  |  | |  |
| *Aramus guarauna* | Agua | Limpkin | | 1, 2, 3, 4, 5, 6, 7, 8, 9, 10, 12, 13, 14, 15, 16, 18, 19, 20, 21, 22 |
| **Rallidae** |  |  | |  |
| *Aramides cajaneus* | Acaj | Gray-necked Wood-Rail | | 9, 11, 13, 14, 15 |
| *Gallinula chloropus* | Gchl | Common Gallinule | | 14 |
| *Porphyrio martinica* | Pmar | Purple Gallinule | | 21 |
| *Porphyrio flavirostris* | Pfla | Azure Gallinule | | 1, 4, 8, 12, 17, 18, 19, 20, 21 |
| **Heliornithidae** |  |  | |  |
| *Heliornis fulica* | Hful | Sungrebe | | 8, 9, 15, 18 |
| **CHARADRIIFORMES** |  |  | |  |
| **Recurvirostridae** |  |  | |  |
| *Himantopus mexicanus* | Hmex | Black-necked Stilt | | 12, 15 |
| **Scolopacidae** |  |  | |  |
| *Calidris fuscicollis* | Cfus | White-rumped Sandpiper | | 15 |
| **Jacanidae** |  |  | |  |
| *Jacana jacana* | Jjac | Wattled Jacana | | 1, 2, 3, 4, 5, 6, 7, 8, 9, 10, 11, 12, 13, 14, 15, 16, 17, 18, 19, 20, 21, 22 |
| **Sternidae** |  |  | |  |
| *Sternula superciliaris* | Ssup | Yellow-billed Tern | | 4, 5, 6, 16, 19, 20, 21, 22 |
| *Phaetusa simplex* | Psim | Large-billed Tern | | 3, 4, 5, 6, 7, 8, 9, 13, 15, 18, 20, 21 |
| **CICONIIFORMES** |  |  | |  |
| **Ciconiidae** |  |  | |  |
| *Ciconia maguari* | Cmag | Maguari Stork | | 21 |
| *Jabiru mycteria* | Jmyc | Jabiru | | 11, 19, 20, 22 |
| *Mycteria americana* | Mame | Wood Stork | | 21 |
| **SULIFORMES** |  |  | |  |
| **Phalacrocoracidae** |  |  | |  |
| *Phalacrocorax brasilianus* | Pbra | Neotropic Cormorant | | 2, 4, 5, 6, 7, 8, 13, 15, 16, 18, 19, 20, 21 |
| **Anhingidae** |  |  | |  |
| *Anhinga anhinga* | Aanh | Anhinga | | 1, 2, 4, 5, 7, 8, 16, 18, 21 |
| **PELECANIFORMES** |  |  | |  |
| **Ardeidae** |  |  | |  |
| *Tigrisoma lineatum* | Tlin | Rufescent Tiger-Heron | | 1, 2, 3, 4, 5, 6, 7, 8, 12, 13, 14, 16, 17, 18, 19, 20, 21, 22 |
| *Ixobrychus exilis* | Iexi | Least Bittern | | 2, 3, 8, 17, 19, 20, 21 |
| *Nycticorax nycticorax* | Nnyc | Black-crowned Night-Heron | | 2, 5, 7, 8, 16, 17, 18, 19, 22 |
| *Butorides striata* | Bstr | Striated Heron | | 1, 2, 3, 4, 5, 8, 9, 10, 12, 13, 15, 16, 17, 18, 19, 20, 21, 22 |
| *Bubulcus ibis* | Bibi | Cattle Egret | | 10, 20, 21 |
| *Ardea cocoi* | Acoc | Cocoi Heron | | 1, 2, 3, 4, 5, 6, 7, 8, 9, 10, 11, 12, 13, 15, 16, 17, 18, 19, 20, 21, 22 |
| *Ardea alba* | Aalb | Great Egret | | 9, 12, 15, 16, 18, 19, 20, 21, 22 |
| *Syrigma sibilatrix* | Ssib | Whistling Heron | | 16 |
| *Egretta thula* | Ethu | Snowy Egret | | 11, 15, 19, 20, 21 |
| **Threskiornithidae** |  |  | |  |
| *Theristicus caudatus* | Tcau | Buff-necked Ibis | | 20 |
| *Platalea ajaja* | Paja | Roseate Spoonbill | | 21 |
| **ACCIPITRIFORMES** |  |  | |  |
| **Accipitridae** |  |  | |  |
| *Busarellus nigricollis* | Bnig | Black-collared Hawk | | 1, 3, 4, 5, 6, 7, 11, 13, 21 |
| *Rostrhamus sociabilis* | Rsoc | Snail Kite | | 1, 2, 4, 5, 6, 7, 9, 13, 15, 18, 19, 20, 21, 22 |
| *Buteogallus urubitinga* | Buru | Great Black Hawk | | 4, 5, 9, 13, 15, 21 |
| **CORACIIFORMES** |  |  | |  |
| **Alcedinidae** |  |  | |  |
| *Ceryle torquata* | Ceto | Ringed Kingfisher | | 1, 2, 3, 4, 5, 7, 8, 9, 10, 13, 15, 18, 19 |
| *Chloroceryle amazona* | Cama | Amazon Kingfisher | | 5, 8, 9, 10, 13, 15, 18, 21, 22 |
| *Chloroceryle americana* | Came | Green Kingfisher | | 1, 2, 5, 8, 9, 10, 12, 18, 21, 22 |
