# Supplementary material for: Comparing species richness, functional diversity and functional composition of waterbird communities along environmental gradients in the neotropics
Source: PLoS One. 2018 Jul 20;13(7):e0200959. doi: 10.1371/journal.pone.0200959 (PMC6054399; doi:10.1371/journal.pone.0200959)
Supplement: S2 Table — (DOCX) [file pone.0200959.s002.docx]

|  | Bodymass | Diet-Inv | Diet-Vend | Diet-Vect | Diet-Vfish | Diet-Vunk | Diet-Scav | Diet-Fruit | Diet-Seed | Diet-PlantO | Dietplasticity | ForStrat-watbelowsurf | ForStrat-wataroundsurf | ForStrat-ground | ForStrat-understory | ForStrat-midhigh | Stratplasticity | Long legged | Hooked bill | Long bill | Swim | Perch |
| --- | --- | --- | --- | --- | --- | --- | --- | --- | --- | --- | --- | --- | --- | --- | --- | --- | --- | --- | --- | --- | --- | --- |
| Sternula.superciliaris | 46.4 | 20 | 0 | 0 | 80 | 0 | 0 | 0 | 0 | 0 | 2 | 100 | 0 | 0 | 0 | 0 | 1 | 0 | 0 | 1 | 0 | 0 |
| Phaetusa.simplex | 235 | 20 | 0 | 0 | 80 | 0 | 0 | 0 | 0 | 0 | 2 | 50 | 30 | 10 | 10 | 0 | 4 | 0 | 0 | 1 | 0 | 0 |
| Calidris.fuscicolis | 43.01 | 80 | 0 | 0 | 0 | 0 | 0 | 0 | 20 | 0 | 2 | 0 | 20 | 80 | 0 | 0 | 2 | 1 | 0 | 0 | 0 | 0 |
| Himantopus.melanurus | 176.82 | 100 | 0 | 0 | 0 | 0 | 0 | 0 | 0 | 0 | 1 | 0 | 100 | 0 | 0 | 0 | 1 | 1 | 0 | 1 | 0 | 0 |
| Phalacrocorax.brasilianus | 1239.29 | 30 | 0 | 20 | 50 | 0 | 0 | 0 | 0 | 0 | 3 | 100 | 0 | 0 | 0 | 0 | 1 | 0 | 1 | 1 | 1 | 0 |
| Anhinga.anhinga | 1235 | 20 | 0 | 20 | 60 | 0 | 0 | 0 | 0 | 0 | 3 | 100 | 0 | 0 | 0 | 0 | 1 | 0 | 0 | 1 | 1 | 1 |
| Chauna.torquata | 4400 | 0 | 0 | 0 | 0 | 0 | 0 | 0 | 20 | 80 | 2 | 0 | 40 | 60 | 0 | 0 | 2 | 0 | 1 | 0 | 0 | 0 |
| Anhuma.cornuta | 3150 | 10 | 0 | 0 | 0 | 0 | 0 | 0 | 0 | 90 | 2 | 0 | 40 | 60 | 0 | 0 | 2 | 0 | 1 | 0 | 0 | 0 |
| Cairina.moschata | 2415.78 | 30 | 0 | 10 | 10 | 0 | 0 | 0 | 20 | 30 | 5 | 0 | 50 | 50 | 0 | 0 | 2 | 0 | 0 | 0 | 1 | 0 |
| Amazonetta.brasiliensis | 499.99 | 20 | 0 | 0 | 0 | 0 | 0 | 40 | 0 | 40 | 3 | 0 | 20 | 80 | 0 | 0 | 2 | 0 | 0 | 0 | 1 | 0 |
| Dendrocygna.viduata | 689.99 | 30 | 0 | 0 | 0 | 0 | 0 | 10 | 20 | 40 | 4 | 80 | 20 | 0 | 0 | 0 | 2 | 0 | 0 | 0 | 1 | 0 |
| Dendrocygna.autumnalis | 755.3 | 10 | 0 | 0 | 0 | 0 | 0 | 0 | 0 | 90 | 2 | 0 | 50 | 50 | 0 | 0 | 2 | 0 | 0 | 0 | 1 | 0 |
| Aramides.sp | 397 | 50 | 0 | 20 | 0 | 0 | 0 | 10 | 10 | 10 | 5 | 0 | 50 | 50 | 0 | 0 | 2 | 1 | 0 | 0 | 0 | 0 |
| Porphyrio.martinica | 235.06 | 30 | 0 | 0 | 0 | 0 | 0 | 0 | 0 | 70 | 2 | 0 | 20 | 30 | 30 | 20 | 4 | 1 | 0 | 0 | 1 | 0 |
| Porphyrio.flavirostris | 92.64 | 50 | 0 | 0 | 0 | 0 | 0 | 0 | 50 | 0 | 2 | 0 | 20 | 60 | 20 | 0 | 3 | 1 | 0 | 0 | 0 | 0 |
| Gallinula.chloropus | 339.63 | 20 | 0 | 10 | 10 | 0 | 0 | 20 | 20 | 20 | 6 | 0 | 20 | 60 | 20 | 0 | 3 | 1 | 0 | 0 | 1 | 0 |
| Jacana.jacana | 106.24 | 60 | 0 | 0 | 0 | 0 | 0 | 0 | 30 | 10 | 3 | 0 | 50 | 50 | 0 | 0 | 2 | 1 | 0 | 0 | 0 | 0 |
| Ardea.cocoi | 1752.44 | 30 | 0 | 30 | 40 | 0 | 0 | 0 | 0 | 0 | 3 | 0 | 100 | 0 | 0 | 0 | 1 | 1 | 0 | 1 | 0 | 0 |
| Ardea.alba | 871.33 | 40 | 10 | 30 | 20 | 0 | 0 | 0 | 0 | 0 | 4 | 0 | 50 | 50 | 0 | 0 | 2 | 1 | 0 | 1 | 0 | 0 |
| Egretta.thula | 371 | 50 | 0 | 30 | 20 | 0 | 0 | 0 | 0 | 0 | 3 | 0 | 70 | 30 | 0 | 0 | 2 | 1 | 0 | 1 | 0 | 0 |
| Bubulcus.ibis | 365.95 | 60 | 10 | 10 | 10 | 0 | 10 | 0 | 0 | 0 | 5 | 0 | 30 | 70 | 0 | 0 | 2 | 1 | 0 | 1 | 0 | 0 |
| Butorides.striata | 201.5 | 30 | 0 | 30 | 40 | 0 | 0 | 0 | 0 | 0 | 3 | 0 | 80 | 20 | 0 | 0 | 2 | 1 | 0 | 1 | 0 | 0 |
| Tigrisoma.lineatum | 812.99 | 70 | 0 | 0 | 0 | 30 | 0 | 0 | 0 | 0 | 2 | 0 | 80 | 20 | 0 | 0 | 2 | 1 | 0 | 1 | 0 | 0 |
| Nycticorax.nycticorax | 810 | 30 | 20 | 30 | 20 | 0 | 0 | 0 | 0 | 0 | 4 | 0 | 50 | 50 | 0 | 0 | 2 | 1 | 0 | 1 | 0 | 0 |
| Ixobrychus.sp | 86.29 | 30 | 0 | 10 | 60 | 0 | 0 | 0 | 0 | 0 | 3 | 0 | 50 | 50 | 0 | 0 | 2 | 1 | 0 | 1 | 0 | 0 |
| Syrigma.sibilatrix | 463 | 70 | 0 | 0 | 0 | 30 | 0 | 0 | 0 | 0 | 2 | 30 | 70 | 0 | 0 | 0 | 2 | 1 | 0 | 1 | 0 | 0 |
| Theristicus.caudatus | 1726 | 70 | 10 | 20 | 0 | 0 | 0 | 0 | 0 | 0 | 3 | 0 | 0 | 100 | 0 | 0 | 1 | 1 | 0 | 1 | 0 | 0 |
| Platalea.ajaja | 1490 | 40 | 0 | 0 | 50 | 0 | 0 | 0 | 0 | 10 | 3 | 0 | 100 | 0 | 0 | 0 | 1 | 1 | 0 | 0 | 0 | 0 |
| Aramus.guarauna | 1080 | 50 | 0 | 20 | 0 | 30 | 0 | 0 | 0 | 0 | 3 | 0 | 50 | 50 | 0 | 0 | 2 | 1 | 0 | 1 | 0 | 0 |
| Jabiru.mycteria | 5996.29 | 10 | 0 | 10 | 80 | 0 | 0 | 0 | 0 | 0 | 3 | 0 | 80 | 20 | 0 | 0 | 2 | 1 | 0 | 1 | 0 | 0 |
| Ciconia.maguari | 3994.99 | 30 | 10 | 20 | 40 | 0 | 0 | 0 | 0 | 0 | 4 | 0 | 80 | 20 | 0 | 0 | 2 | 1 | 0 | 1 | 0 | 0 |
| Mycteria.americana | 2554.47 | 20 | 0 | 10 | 70 | 0 | 0 | 0 | 0 | 0 | 3 | 0 | 50 | 50 | 0 | 0 | 2 | 1 | 0 | 1 | 0 | 0 |
| Buteogallus.urubitinga | 1152.87 | 20 | 20 | 30 | 10 | 0 | 10 | 10 | 0 | 0 | 6 | 0 | 0 | 40 | 10 | 50 | 3 | 0 | 1 | 0 | 0 | 1 |
| Rostrhamus.sociabilis | 366.94 | 100 | 0 | 0 | 0 | 0 | 0 | 0 | 0 | 0 | 1 | 0 | 50 | 50 | 0 | 0 | 2 | 0 | 1 | 0 | 0 | 1 |
| Busarellus.nigricollis | 766.14 | 10 | 0 | 0 | 90 | 0 | 0 | 0 | 0 | 0 | 2 | 0 | 50 | 50 | 0 | 0 | 2 | 0 | 1 | 0 | 0 | 1 |
| Heliornis.fulica | 132 | 30 | 0 | 30 | 20 | 0 | 0 | 0 | 20 | 0 | 4 | 0 | 70 | 30 | 0 | 0 | 2 | 0 | 0 | 0 | 1 | 0 |
| Ceryle.torquata | 317 | 20 | 0 | 20 | 60 | 0 | 0 | 0 | 0 | 0 | 3 | 90 | 0 | 10 | 0 | 0 | 1 | 0 | 0 | 1 | 0 | 1 |
| Chloroceryle.americana | 33.73 | 40 | 0 | 0 | 60 | 0 | 0 | 0 | 0 | 0 | 2 | 70 | 10 | 10 | 10 | 0 | 4 | 0 | 0 | 1 | 0 | 1 |
| Chloroceryle.amazona | 126.38 | 50 | 0 | 0 | 50 | 0 | 0 | 0 | 0 | 0 | 2 | 100 | 0 | 0 | 0 | 0 | 1 | 0 | 0 | 1 | 0 | 1 |
