# Supplementary material for: Comparing species richness, functional diversity and functional composition of waterbird communities along environmental gradients in the neotropics
Source: PLoS One. 2018 Jul 20;13(7):e0200959. doi: 10.1371/journal.pone.0200959 (PMC6054399; doi:10.1371/journal.pone.0200959)
Supplement: S3 Table — AICc = Akaike Information Criteria corrected for small sample sizes. Model terms are coded as: 1, Area (ha); 2, AVdiv = aquatic vegetation diversity index; 3, Floating; 4, Emergent. df: Degrees of freedom. (DOCX) [file pone.0200959.s003.docx]

S2 Table. Top-ranked candidate models explaining variation in waterbird species richness in the floodplain lakes. AICc = Akaike Information Criteria corrected for small sample sizes. Model terms are coded as: 1, Area (ha); 2, AVdiv = aquatic vegetation diversity index; 3, Floating; 4, Emergent. df: Degrees of freedom.

| Model | df | Log-likelihood ratio | AICc | ∆AICc | AICc weight |
| --- | --- | --- | --- | --- | --- |
| 1, 2, 3 | 5 | -55.65 | 125.06 | 0.00 | 0.32 |
| 1, 2, 4 | 5 | -55.81 | 125.36 | 0.31 | 0.27 |
| 1, 2 | 4 | -57.76 | 125.87 | 0.81 | 0.21 |
| 1, 4 | 4 | -57.78 | 125.92 | 0.87 | 0.20 |
