# Supplementary material for: Comparing species richness, functional diversity and functional composition of waterbird communities along environmental gradients in the neotropics
Source: PLoS One. 2018 Jul 20;13(7):e0200959. doi: 10.1371/journal.pone.0200959 (PMC6054399; doi:10.1371/journal.pone.0200959)
Supplement: S4 Table — AICc = Akaike Information Criteria corrected for small sample sizes. Model terms are coded as: 1, Emergent; 2, Grass; 3, Mdepth.margin; 4, NoVeg; 5, Transparency; 6, VCdepth.margin (variation coefficient of margin depth). df: Degrees of freedom. (DOCX) [file pone.0200959.s004.docx]

S3 Table. Top-ranked candidate models explaining variation in waterbird FDis in the floodplain lakes. AICc = Akaike Information Criteria corrected for small sample sizes. Model terms are coded as: 1, Emergent; 2, Grass; 3, Mdepth.margin; 4, NoVeg; 5, Transparency; 6, VCdepth.margin (variation coefficient of margin depth). df: Degrees of freedom.

| Model | df | Log-likelihood ratio | AICc | ∆AICc | AICc weight |
| --- | --- | --- | --- | --- | --- |
| 1, 2, 3, 6 | 6 | 51.59 | -85.58 | 0.00 | 0.42 |
| 1, 2, 3, 5, 6 | 7 | 53.13 | -84.27 | 1.31 | 0.22 |
| 2, 3, 4, 6 | 6 | 50.84 | -84.09 | 1.49 | 0.20 |
| 2, 3, 6 | 5 | 48.70 | -83.65 | 1.93 | 0.16 |
